# Functional Expression of Parasite Drug Targets and Their Human Orthologs in Yeast

**DOI:** 10.1371/journal.pntd.0001320

**Published:** 2011-10-04

**Authors:** Elizabeth Bilsland, Pınar Pir, Alex Gutteridge, Alexander Johns, Ross D. King, Stephen G. Oliver

**Affiliations:** 1 Department of Biochemistry and Cambridge Systems Biology Centre, University of Cambridge, Cambridge, United Kingdom; 2 Department of Computer Science, Aberystwyth University, Aberystwyth, Ceredigion, United Kingdom; McGill University, Canada

## Abstract

**Background:**

The exacting nutritional requirements and complicated life cycles of parasites mean that they are not always amenable to high-throughput drug screening using automated procedures. Therefore, we have engineered the yeast *Saccharomyces cerevisiae* to act as a surrogate for expressing anti-parasitic targets from a range of biomedically important pathogens, to facilitate the rapid identification of new therapeutic agents.

**Methodology/Principal Findings:**

Using pyrimethamine/dihydrofolate reductase (DHFR) as a model parasite drug/drug target system, we explore the potential of engineered yeast strains (expressing DHFR enzymes from *Plasmodium falciparum*, *P. vivax*, *Homo sapiens*, *Schistosoma mansoni*, *Leishmania major*, *Trypanosoma brucei* and *T. cruzi*) to exhibit appropriate differential sensitivity to pyrimethamine. Here, we demonstrate that yeast strains (lacking the major drug efflux pump, Pdr5p) expressing yeast (*^Sc^DFR1*), human (*^Hs^DHFR*), *Schistosoma* (*^Sm^DHFR*), and *Trypanosoma* (*^Tb^DHFR* and *^Tc^DHFR*) DHFRs are insensitive to pyrimethamine treatment, whereas yeast strains producing *Plasmodium* (*^Pf^DHFR* and *^Pv^DHFR*) DHFRs are hypersensitive. Reassuringly, yeast strains expressing field-verified, drug-resistant mutants of *P. falciparum DHFR* (*^Pf^dhfr* ^51I,59R,108N^) are completely insensitive to pyrimethamine, further validating our approach to drug screening. We further show the versatility of the approach by replacing yeast essential genes with other potential drug targets, namely phosphoglycerate kinases (PGKs) and N-myristoyl transferases (NMTs).

**Conclusions/Significance:**

We have generated a number of yeast strains that can be successfully harnessed for the rapid and selective identification of urgently needed anti-parasitic agents.

## Author Summary

Parasites kill millions of people every year and leave countless others with chronic debilitating disease. These diseases, which include malaria and sleeping sickness, mainly affect people in developing countries. For this reason, few drugs have been developed to treat them. To make matters worse, many parasites are developing resistance to the drugs that are available. Thus, there is an urgent need to develop new drugs, but this is hampered by the fact that most parasites are difficult or impossible to grow in the laboratory. To address this, we have engineered baker's yeast to be dependent on the function of enzymes from either parasites or humans. In all, our engineered yeast constructs encompass six parasites (causing malaria, schistosomiasis, leishmaniasis, sleeping sickness, and Chagas disease) and three different enzymes that are known or potential drug targets. Further, we have increased yeast's sensitivity to drugs by deleting the gene for its major drug efflux pump. Because yeast is robust and easy to grow in the laboratory, we can use a robot to screen for drugs that will kill yeast dependent on a parasite enzyme, but not touch yeast dependent on the equivalent human enzyme.

## Introduction

Parasitic diseases such as malaria, schistosomiasis, leishmaniasis, sleeping sickness, and Chagas disease affect millions of people every year, leading to severe morbidity and death. For example malaria, caused by parasites of the genus *Plasmodium*, kills 1–3 million people every year (http://www.who.int/mediacentre/factsheets/fs094/en/index.html). The disease is primarily treated by chloroquine, artemisinin, and antifolates (e.g. pyrimethamine). However, *Plasmodium* spp. have become resistant to all of these drugs [1]. Schistosomiasis, caused by the blood fluke *Schistosoma*, is the second most important parasitic disease worldwide, affecting *ca*. 207 million people (http://www.who.int/mediacentre/factsheets/fs115/en/index.html) [2], and causing the death of greater than 300,000 individuals per annum [3]. Schistosomiasis is commonly treated through administration of praziquantel, but the sole use of this drug against all human infective schistosome species raises the concern that drug-resistant parasites may develop [4]. Sleeping sickness (or African trypanosomiasis), caused by *Trypanosoma brucei*, infects about 300,000 people each year leading to about 40,000 deaths [5], [6]. Chagas disease, caused by *T.cruzi*, is endemic to Latin America, where *ca*. 17 million people are infected, leading to about 21,000 deaths reported each year [7]. Very few drugs are available for the treatment of patients infected with *Trypanosoma* spp. and none is satisfactory due to low efficacy, high cost, or unacceptable side effects [8], [9], [10]. Leishmaniasis, caused by *Leishmania* spp. is endemic in 88 countries affecting 12 million people (http://www.who.int/leishmaniasis/en/). It is traditionally treated with antimony compounds, but resistance to this class of drugs is increasing and very few novel drugs are under development. All of this demonstrates that the design or discovery of novel antiparasitic drugs is a global imperative.

For the past 30 years, the yeast *Saccharomyces cerevisiae* has been used successfully as a vehicle for the expression of heterologous proteins with the aim of understanding their function, producing high levels of recombinant protein, or studying the effect of drugs on defined targets (for review see [11]). By performing genome-wide drug sensitivity screens (chemogenomic profiling) [12], [13], [14], [15], [16], [17] of yeast mutants with the antimalarials quinine [18], St. John's Wort [19] and artemisinin [20], researchers were able to identify their primary targets as well as identify potential side effects. Furthermore, several groups have been able to complement yeast loss-of function mutations by expressing coding sequences from parasites such as *Plasmodium* [21], [22], *Schistosoma* [23], [24], [25], *Leishmania* [26] or *Trypanosoma* [6], [27], [28], [29], [30], [31].

Yeast cells expressing parasite proteins potentially provide a well-characterised platform for functional studies of heterologous proteins as well as for screens attempting to identify novel drugs including antiparasitics [32], [33], [34], [35]. For instance, Geary and co-workers expressed potential drug targets for *Haemonchus contortus* (wireworm, an important parasite of ruminants) in *Escherichia coli* [36], [37] and later in *Saccharomyces cerevisiae* [38], and pioneered the development of high-throughput drug screens for antiparasitics in yeast [38].

A well-characterised anti-parasitic drug target is dihydrofolate reductase (DHFR). DHFR is the enzyme responsible for converting dihydrofolate into tetrahydrofolate, an intermediate in the synthesis of purines, thymidylic acid, and certain amino acids (e.g. methionine). DHFR is present in organisms ranging from bacteria to humans and is the target of pyrimethamine treatment of malaria and human tumours, since rapidly growing cells require folate to produce thymine [39]. Sibley and co-workers [22], [40], [41] have done extensive work on the complementation of yeast *dfr1* mutations by overexpression of human and *Plasmodium DHFRs* and demonstrated the suitability of the strains for drug screens in plate assays.

Phosphoglycerate kinase (PGK) is a central enzyme in glycolysis and gluconeogesis; it catalyzes the transfer of high-energy phosphoryl groups from the acyl phosphate of 1,3-bisphosphoglycerate to ADP to produce ATP. PGKs are essential for the blood stages of parasites but the human enzyme is not expressed in erythrocytes; therefore, the enzyme has been proposed as a drug target [42], [43].

N-myristoyltransferase (NMT) is an enzyme responsible for the co- and post-translational modification of proteins by transferring myristate groups to N-terminal glycine residues, allowing their targeting to various membranes [5], [44]. NMTs are essential enzymes conserved from kinetoplastid parasites to humans and have been successfully demonstrated as drug targets [5].

In spite of the pressing need for new treatments targeting neglected diseases, pharmaceutical companies have had little interest in the research and development of new medicines towards diseases affecting overwhelmingly or exclusively developing countries [45], [46]. Thankfully, with the investment of funds from organizations such as the Bill and Melinda Gates Foundation, Medicines for Malaria Venture (MMV), the Drugs for Neglected Diseases initiative (DNDi), and the Institute for One World Health (IOWH) this scenario is changing [47], [48]. Now, pharmaceutical giants such as Novartis [49], GSK [50], Pfizer (just to name a few) investing great efforts in the development of novel antimalarials (www.mmv.org/research-development/science-portfolio). However, due to the diversity of parasite species and their complex life cycles, the development of inexpensive and rapid drug screening methods is a constant challenge. Various groups have developed efficient high-throughput drug screening methods based on intact parasites [51], [52], [53], [54]. However, these are specific to one or a few parasite species and do not always provide information concerning the target of the hit compound. Conversely, the standard alternative of using pure proteins as targets can be unsatisfactory because the assay neglects all other biological interactions of the candidate compounds [35]. To meet this challenge, we have developed a series of yeast strains that can be used to screen for drugs against multiple drug targets from multiple parasites with a single experimental set-up.

## Methods

### Construction of plasmid maps and phylogenetic trees

We constructed plasmid maps and Genbank files of the constructs expressing heterologous protein using the program CLC Genomics Workbench. We translated the coding regions of each of the proteins and performed protein alignments of them against *Saccharomyces cerevisiae* Dfr1p, Nmt1p and Pgk1p. We constructed the similarity tree using the standard settings from CLC Genomics Workbench (neighbor-joining, bootstrap analysis, 100 replicates).

### Strain and plasmid constructs

The DHFR/PGK/NMT-coding regions of *Schistosoma mansoni* (*Sm*), *Saccharomyces cerevisiae* (*Sc*), *Leishmania major* (*Lm*), *Trypanosoma cruz*i (*Tc*) and *T.brucei* (*Tb*) were PCR amplified from genomic DNA templates and cloned into pCM188 [55]. The following plasmids were constructed: pCM*^Sm^DHFR* (pCM188 with the complete open reading frame of *Schistosoma mansoni* DHFR under the control of the tetracycline regulatable promoter TetO2), pCM*^Sc^DHFR*, pCM*^Lm^DHFR*, pCM*^Tc^DHFR*, pCM*^Tb^DHFR*, pCM*^Sm^PGK*, pCM*^Lm^PGKB*, pCM*^Tc^PGK*, pCM*^Tb^PGK*, pCM*^Sm^NMT*, pCM*^Lm^NMT*, pCM*^Tc^NMT*, pCM*^Tb^NMT* (Table S1).

Human (*Hs*) *PGK* and *NMT2* were PCR amplified from a human cerebellum cDNA library and cloned into pCM188 to produce pCM*^Hs^PGK* and pCM*^Hs^NMT2* (Table S1).

The DHFR coding sequences from human, *Plasmodium falciparum* (*Pf*), and *P.vivax* (*Pv*), as well as PGK and NMT from *P.vivax*, were synthesised by GENEART with a codon usage suitable for expression in yeast. These synthetic constructs were sub-cloned into pCM188 to generate pCM*^Hs^DHFR*, pCM*^Pf^DHFR*, pCM*^Pv^DHFR*, pCM*^Pv^PGK* and pCM*^Pv^NMT* (Table S1).

The DHFR mutations N51I, C59R and S108N confer resistance to antifolates in wild *Plasmodium falciparum* populations; we designate such resistant alleles by the preceding superscript *Pfr* and give the amino-acid changes in parentheses in a succeeding superscript. Two rounds of site-directed mutagenesis were performed to introduce these mutations into pCM*^Pf^DHFR* to generate pCM*^PfR^dhfr* ^(51I,59R,108N)^ (Table S1). All constructs were verified by sequencing (Figures S1, S2, S3, S4, S5, S6, S7, S8 and Text S1, S2, S3, S4, S5, S6, S7, S8, S9, S10, S11, S12, S13, S14, S15, S16, S17, S18, S19, S20, S21).

Deletions of the yeast *PDR5* coding sequence (specifying the major drug efflux pump) from *dfr1*, *pgk1* and *nmt1* heterozygous mutant strains were performed as previously described [56]. pCM*-DHFR* constructs were transformed into *dfr1*Δ*::KanMX/DFR1 pdr5*Δ*::HisMX/PDR5* strains (BY4743 background [57]). pCM*-PGK* constructs were transformed into *pgk1*Δ*::KanMX/PGK1 pdr5*Δ*::HisMX/PDR5* strains (BY4743 background). pCM*-NMT* constructs were transformed into *nmt1*Δ*::KanMX/NMT1 pdr5*Δ*::HisMX/PDR5* strains (BY4743 background). Heterozygous diploid strains were then sporulated and dissected using a micromanipulator (Singer MSM). Derived haploids with the genotype *dfr1*Δ*::KanMx MET15 lys2*Δ*0 MATα*, *pgk1*Δ*::KanMx MET15 lys2*Δ*0 MATα* or *nmt1*Δ*::KanMx met15 LYS2 MATa* were selected for drug screens (Table S2).

### Growth conditions

Standard growth conditions and either YPD (2% peptone, 1% yeast extract, 2% glucose) or YNB-glucose (0.68% yeast nitrogen base, 2% ammonium sulphate, 2% glucose) with the relevant supplements were used for all assays.

### Liquid growth assays and maximum growth rate calculations

Cultures of wild type (BY4741) and transformant yeast strains were inoculated into 200 µl of YPD in the wells of 96-well microtiter plates and grown for 40 hours at 30°C. Growth was monitored with the BMG Optima multiplate reader. Each sample was present in quadruplicates, distributed randomly throughout the plate and OD_595_ measurements were made ever 10 minutes. A 384-well plate (with 70 µl of YPD + doxycycline) was prepared in a similar manner.

The OD readings from the plate reader were log-transformed and 7 consecutive readings were used to calculate exponential growth rate of the culture in each well. These exponential growth rates were then normalized by dividing by the average exponential growth rate of the wild type culture grown on the same plate. Normalized growth rates were averaged across the 4 or 5 replicates and standard deviations calculated.

### Plate assays for drug sensitivity

Serial dilutions (5x) of stationary phase cultures were prepared in 96-well plates and replicated onto agar plates manually or in quadruplicate onto agar plates using a robot (Singer RoToR). Cells were allowed to grow for 2 days at 30°C.

### Quantification of growth on agar plates

Strains were spotted in four replicates (Figure S10). Each pair of rows on the image corresponds to a strain, spotted in increasing dilutions (1∶1, 1∶5, 1∶25 …) in blocks of four. The images of the plates were produced in Gel Doc 2000 (BioRad) and saved as .jpg files. Matlab was used to convert the images to three-dimensional matrices, the first and second dimensions were the vertical and horizontal dimensions of the image and third dimension was the colour channel in RBG 24-bit format. As the images were saved in grey scale, choice of channel did not make a major impact on quantification of colonies; third channel (Green) was used.

The average intensities of pixels on each column and row in the image were calculated, intersection points of rows and columns with highest average intensity among their neighbours (in a window of 5×5) were set as the spot or colony centres. Once the colony centres were set, a 16 pixels×16 pixels diamond shape colony frame was set around each colony centre, and average intensity was calculated for each colony frame. Then the pixels with 50% larger intensity than the average of the colony frame were counted and recorded as the colony size.

## Results

### Defining the drug targets

We have defined our candidate drug targets based on the following criteria: (i) the target should be an enzyme that is essential in yeast (this permits verification of the functional expression of the target); (ii) the target should be essential, or predicted to be essential, in most parasites; (iii) it may be present in human (a ‘humanized’ yeast strain will be used as a control in the screens); (iv) there should be a low similarity between human and parasite proteins; (v) the target should be one suggested by the TDR targets database (http://tdrtargets.org/). Combining these criteria, we selected 4 drug targets for expression in yeast: dihydrofolate reductase (DHFR, EC:1.5.1.3), phosphoglycerate kinase (PGK, EC:2.7.2.3), N-myristoyl transferase (NMT, EC:2.3.1.97), and farnesyl pyrophosphate synthetase (FPS, EC:2.5.1.1, EC:2.5.1.10). As explained in the Introduction, all of these enzymes have previously been proposed as useful targets for antiparasitic drugs, and many have been used in drug screens [5], [22], [41], [42], [43], [44], [58].

### Complementation

**Figure 1:**
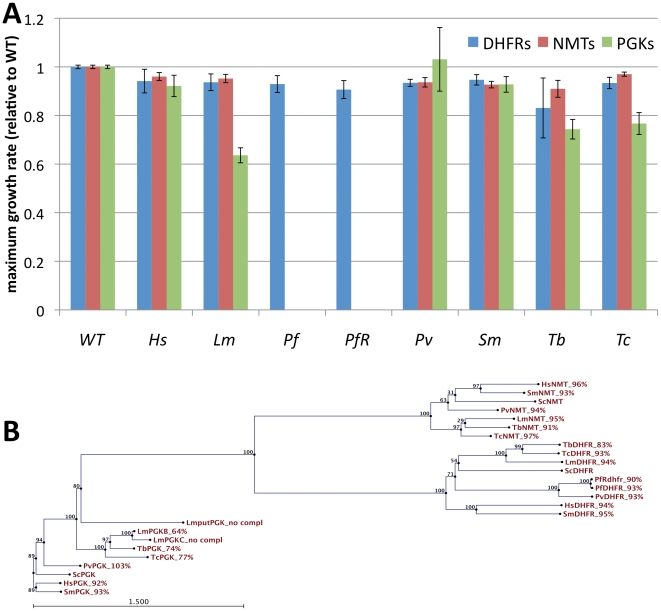
Complementation of yeast *dfr1*, *nmt1* and *pgk1* deletion mutants with human and parasite orthologs. A) Maximum growth rates of yeast strains expressing cds for heterologous dihydrofolate reductases (DHFRs), N-myristoyl transferases (NMTs) or phosphoglycerate kinases (PGKs) from human (Hs), *Leishmania major* (Lm), *Plasmodium falciparum* (Pf and PfR), *P.vivax* (Pv), *Schistosoma mansoni* (Sm), *Trypanosoma brucei* (Tb) or *T. cruzi* (Tc). B) Phylogenetic tree and maximum growth rate (% of wild-type growth) of the heterologous NMTs, DHFrs and PGKs. All strains are *pdr5Δ* mutants.

We have constructed a series of four plasmids containing the coding sequences (cds) for human DHFR, PGK, NMT, and FPS and transformed these into diploid yeast strains that are heterozygous deletion mutants for the gene encoding the corresponding essential enzyme, namely: *DFR1* (*Sc*DHFR), *PGK1* (*Sc*PGK), *NMT1* (*Sc*NMT) or *ERG20* (*Sc*FPS). The transformed diploids were then sporulated. Two of the four haploid spores in the tetrad will carry the deletion for the essential yeast gene and will only grow if that mutation is complemented by the orthologous human cds. We observed that the overexpression of the cds for human DHFR (a synthetic gene codon-optimised for expression in yeast was used), PGK or NMT2 could complement the essential function of the yeast deletions (Figure 1), whereas human FPS could not complement the deletion of the yeast FPS (data not shown). We then constructed plasmids expressing codon-optimized cds for *Plasmodium vivax* DHFR, NMT and PGK, *P. falciparum* DHFR and drug resistant *P. falciparum* dhfr, and verified that these heterologous sequences could also complement the yeast deletions. We constructed strains expressing cds for *Schistosoma mansoni*, *Leishmania major*, *Trypanosoma cruzi* and *T. brucei* DHFRs and NMTs, and found that each of them could complement yeast deletions, albeit with varying efficiencies (Figure 1). The same was true for each of the *Schistosoma mansoni*, *Trypanosoma cruzi*, and *T. brucei* PGKs tested. We tested whether cds for the different *Leishmania major* PGKs – the cytosolic PGKB, the glycosomal PGKC, and the putative PGK LmjF30.3380 - could complement a yeast *pgk1* deletion mutation. We found that only PGKB could complement the yeast deletion and resulted in a slow-growth phenotype (approximately 64% of the maximum growth rate of a wild-type strain) even with full expression of the heterologous protein (Figure 1).

Plasmid maps for all of these plasmids are shown in Figures S1, S2, S3, S4, S5, S6, S7, S8 and the full sequences of the plasmids can also be found in the Supplementary Material (Text S1, S2, S3, S4, S5, S6, S7, S8, S9, S10, S11, S12, S13, S14, S15, S16, S17, S18, S19, S20, S21).

### Modulated expression

We cloned cds for all of the heterologous proteins into pCM188 plasmids, under the control of a TetO2 promoter, to allow the modulated expression of each drug target in yeast. TetO2 is a powerful constitutive promoter but can be repressed by the addition of a tetracycline analogue (doxycycline) to the growth medium. Thus, normal growth conditions result in the full expression on the cloned cds;, however, by adding doxycycline, we can reduce the expression of the parasite or human cds to levels that should be inversely proportional to the concentration of doxycycline added . We measured the growth rate of the parasite mimetic or human mimetic yeast strains in the presence of 2, 5, 10 or 20 mg/L doxycycline and found that the reduction in growth rate of yeast expressing human or parasite cds for either DHFR or NMT were relatively insensitive to the addition of doxycline (≤ 25%) growth-rate inhibition (Figure S9). In contrast, addition of doxycline to strains express cds for PGK displayed up to 75% growth-rate upon doxycycline treatment (Figure S9). While these results indicate that only small amounts of the heterologous enzymes are often sufficient to achieve full complementation of the yeast deletion mutation, we demonstrate below that reduction in enzyme concentration achieved by inhibiting promoter activity with doxycycline is sufficient to enhance the sensitivity of yeast growth to the action of drugs that inhibit the activity of target enzymes to a useful extent.

### Plate assays

**Figure 2:**
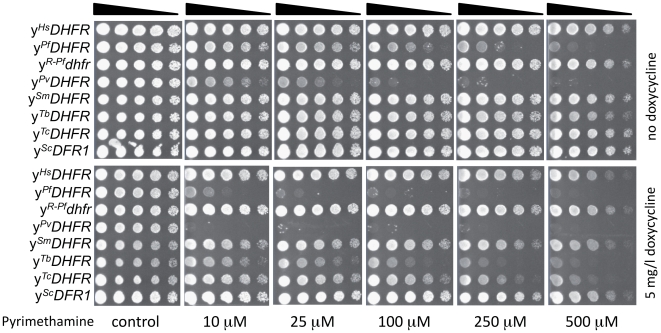
Pyrimethamine sensitivity of yeast strains expressing heterologous dihydrofolate reductases (DHFRs). Serial dilutions (5x) of yeast cultures expressing cds for human (Hs), *Plasmodium falciparum* (Pf), drug resistant *P. falciparum* (PfR), *P.vivax* (Pv), *Schistosoma mansoni* (Sm), *Trypanosoma brucei* (Tb), *T. cruzi* (Tc) or *Saccharomyces cerevisiae* (Sc) DHFRs spotted onto agar plates containing pyrimethamine at the stated doses with or without doxycycline (5 mg/L).

PGKs and NMTs are fairly novel drug targets and therefore we had no access to specific inhibitors for the different species tested. However, *Plasmodium* DHFRs are classic drug targets, which can be specifically inhibited by the antimalarial drug pyrimethamine. Therefore, we performed drug sensitivity assays (on agar plates) of the different yeast transformants expressing parasite mimetic or human DHFRs in the presence of various concentrations of pyrimethamine. We found that yeast strains expressing *Plasmodium vivax* DHFR (*y^Pv^DHFR*) showed sensitivity to concentrations of pyrimethamine ranging from 10 to 500 µM (Figure 2, upper panels). Yeast strains expressing *Plasmodium falciparum* DHFR (*y^Pf^DHFR*) showed sensitivity to concentrations of pyrimethamine ranging from 100 to 500 µM (Figure 2, upper panels). No other strain showed sensitivity to the pyrimethamine concentrations tested. Importantly, by modulating the expression of the heterologous cds by adding 5 mg/L doxycycline to the growth media, it was possible to increase the sensitivity of the strains to pyrimethamine by approximately 50-fold (Figure 2, lower panels).

### Effect of a *pdr5* deletion on pyrimethamine sensitivity

**Figure 3:**
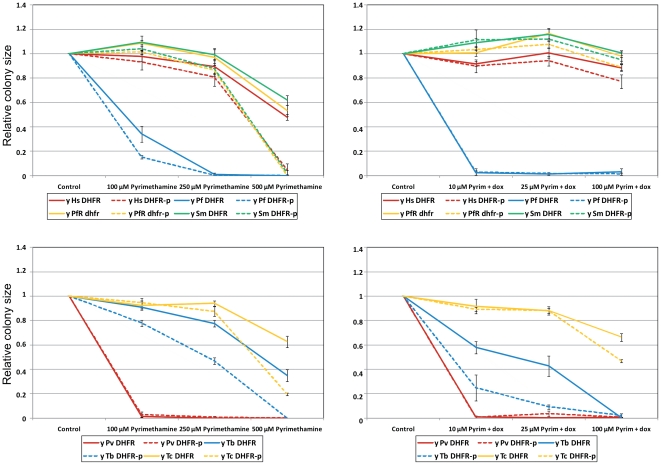
Effect of the drug efflux pump Pdr5p on sensitivity to pyrimethamine. Graphs showing the quantification of growth on agar plates (containing the indicated concentrations of pyrimethamine +/- 5 mg/L of doxycycline) of yeast strains expressing cds for heterologous DHFRs. Solid lines: *PDR5* strains: dashed lines: *pdr5Δ* mutants.

With the aim of further increasing the sensitivity of our assays, we deleted the *PDR5* gene, which encodes the major yeast multidrug export pump, from all of our strains and tested the effect of such a deletion on the pyrimethamine sensitivity of strains expressing heterologous DHFRs. We spotted (using a Singer RoTor robot) serial dilutions of *PDR5* and *pdr5Δ* strains onto rectangular agar plates containing a series of concentrations of pyrimethamine with or without doxycycline (5 mg/L). Following quantification of the data (Figure S10), we observed that removal of the multidrug export pump by the *pdr5Δ* deletion significantly increased the strains' sensitivity to pyrimethamine, such that (in the presence of doxycycline) the sensitivity of the *Trypanosoma brucei* DHFR could be observed (Figure 3).

## Discussion

Yeast cells are suitable hosts for the expression of heterologous proteins from various species, including enzymes essential to the different life-cycle stages of parasites. Some of these parasite life stages cannot be propagated under laboratory conditions, and thus yeast provides a practical and flexible platform for *in vivo* drug screens. Here, we have reported the construction of a series of yeast strains that are identical apart from the cds for different heterologous drug targets (*DHFR*, *PGK* or *NMT*) which they express. For all of these targets, we also constructed yeast strains expressing the cds for the equivalent human enzyme, thus permitting the design of screens for agents that discriminate between the parasite and human targets.

Sibley and co-workers [22], [40], [41] have done extensive work on the complementation of yeast *dfr1* mutations by overexpression of human and *Plasmodium DHFRs* and demonstrated the suitability of yeast strains for drug screens in plate assays. Now, we have demonstrated that the *DHFR* cds from *Schistosoma mansoni*, *Leishmania major*, *Trypanosoma cruzi* and *T. brucei* can also successfully complement a *dfr1* null mutant of yeast. Furthermore, we have found that yeast cells expressing *T. brucei* DHFR are partially sensitive to pyrimethamine (Figure 2) and that strains expressing *T. cruzi* or *S. mansoni* DHFRs are sensitive to the chemotherapeutic agent methotrexate (data not shown). In addition, we have also successfully complemented yeast deletion mutants with cds for two new potential drug targets from parasites: N-myristoyl transferase (NMT) and phosphoglycerate kinase (PGK). To the best of our knowledge, this is the first evidence for the functional expression of these parasite enzymes in yeast. While all of the data presented in this paper relate to screens against yeast cells spotted onto agar plates, we have confirmed all the results for DHFR using yeast strains grown in liquid cultures in 384-well microtiter trays. Indeed, we intend to use such liquid cultures in HTP screens based on the series of yeast strains reported here.

Yeast cells may be refractory to chemotherapeutic drugs due to their thick cell walls and the high levels of expression of multiple drug efflux pumps. Thus the genetic manipulation of yeast strains for use in screens in order to increase their drug sensitivity is highly desirable. We have improved the drug sensitivity of our yeast constructs in two ways. First, all cds encoding heterologous enzymes that represent drug targets were placed under control of the TetO2 promoter, so that their expression could be down-regulated by addition of the tetracycline analogue, doxycycline. We demonstrated that additions of doxycycline to the growth medium enhanced the pyrimethamine sensitivity of parasite DHFR enzymes expressed in yeast (Figure 2). Second, we demonstrated (again using DHFR and pyrimethamine) that the deletion of the gene *PDR5*, which encodes the major yeast ABC transporter, can increase the sensitivity of drug screens (Figure 3). Moreover the use of *pdr5Δ* mutants in conjunction with pyrimethamine has a synergistic effect on drug sensitivity (Figure 3). The ease of genetically manipulation in yeast should allow further improvements in drug sensitivity in the future; for instance, by modifying the sequence or expression of genes (such *CWP1, CWP2*, or *PSA1*) that encoding cell wall proteins [59].

We believe that many more enzyme targets and parasite species can be studied using a similar approach and are currently employing some of our strains in high-throughput drug screens using a Robot Scientist [60], [61], [62] in search of novel antiparasitic agents.

## Supporting Information

Figure S1 **Map of pCM188.** Plasmid backbone used for cloning of the cds for heterologous DHFRs, NMTs and PGKs under the control of the tetracycline-regulatable promotor: TetO2.
(TIF)

Figure S2 **Maps of the** ***Plasmodium falciparum*** **complementation plasmids.** Plasmids for expression of cds for heterologous wild-type *Plasmodium falciparum* DHFR (PfDHFR) and the drug-resistant *Plasmodium falciparum* DHFR (PfRdhfr) under the control of the TetO2 promoter.
(TIF)

Figure S3 **Maps of the** ***Plasmodium vivax*** **complementation plasmids.** Plasmids for expression of cds for heterologous *Plasmodium vivax* DHFR, NMT and PGK under the control of the TetO2 promoter.
(TIF)

Figure S4 **Maps of the** ***Schistosoma mansoni*** **complementation plasmids.** Plasmids for expression of cds for heterologous *Schistosoma mansoni* DHFR, NMT and PGK under the control of the TetO2 promoter.
(TIF)

Figure S5 **Maps of the** ***Trypanosoma brucei*** **complementation plasmids.** Plasmids for expression of cds for heterologous *Trypanosoma brucei* DHFR, NMT and PGK under the control of the TetO2 promoter.
(TIF)

Figure S6 **Maps of the** ***Trypanosoma cruzi*** **complementation plasmids.** Plasmids for expression of cds for heterologous *Trypanosoma cruzi* DHFR, NMT and PGK under the control of the TetO2 promoter.
(TIF)

Figure S7 **Maps of the human complementation plasmids.** Plasmids for expression of cds for heterologous *Homo sapiens* DHFR, NMT2 and PGK under the control of the TetO2 promoter.
(TIF)

Figure S8 **Maps of the** ***Leishmania major*** **complementation plasmids.** Plasmids for expression of cds for heterologous *Leishmania major* DHFR, NMT and PGKB under the control of the TetO2 promoter.
(TIF)

Figure S9 **Effect of doxycycline on the maximum growth rate of yeast strains expressing heterologous drug targets.** Maximum growth rates (relative to that of the wild type) of yeast strains expressing cds for human or parasite DHFRs, NMTs or PGKs under the control of the TetO2 promoter.
(TIF)

Figure S10
**Quantification of the effect of** ***pdr5Δ*** **mutations on pyrimethamine sensitivity**. A) Example of plate where serial dilutions of yeast cultures expressing cds for heterologous DHFRs were spotted in quadruplicate onto agar plates containing pyrimethamine and doxycycline. Wild-type *PDR5* strains were spotted on the left half of the plate and *pdr5Δ* deletion mutants on the right half of the plate. B) The intercepts of the brightest row and column of the spots were marked as `colony center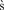. C) The area enclosed by a diamond-shaped frame around the colony center was set as the `colony windo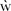. D) Number of pixels brighter than the threshold within the colony window was set as the `colony sizè, which corresponds to spot's total area.(TIF)

Table S1 **Plasmids used in this study.** Details and source of the complementation plasmids used in this work.
(DOC)

Table S2 **Strains used in this study.** Details and source of strains used in this work.
(DOC)

Text S1 **pCM188.** Sequence and features of the plasmid backbone used for cloning of the heterologous DHFRs, NMTs and PGKs under the control of the tetracycline-regulatable promoter: TetO2.
(GBK)

Text S2 **pCMPfDHFR.** Sequence and features of the *Plasmodium falciparum* DHFR complementation plasmid.
(GBK)

Text S3 **pCMPfRdhfr.** Sequence and features of the drug-resistant *Plasmodium falciparum* DHFR complementation plasmid.
(GBK)

Text S4 **pCMPvDHFR.** Sequence and features of the *Plasmodium vivax* DHFR complementation plasmid.
(GBK)

Text S5 **pCMSmDHFR.** Sequence and features of the *Schistosoma mansoni* DHFR complementation plasmid.
(GBK)

Text S6 **pCMTbDHFR.** Sequence and features of the *Trypanosoma brucei* DHFR complementation plasmid.
(GBK)

Text S7 **pCMTcDHFR.** Sequence and features of the *Trypanosoma cruzi* DHFR complementation plasmid.
(GBK)

Text S8 **pCMHsDHFR.** Sequence and features of the human DHFR complementation plasmid.
(GBK)

Text S9 **pCMLmDHFR.** Sequence and features of the *Leishmania major* DHFR complementation plasmid.
(GBK)

Text S10 **pCMPvNMT.** Sequence and features of the *Plasmodium vivax* NMT complementation plasmid.
(GBK)

Text S11 **pCMSmNMT.** Sequence and features of the *Schistosoma mansoni* NMT complementation plasmid.
(GBK)

Text S12 **pCMTbNMT.** Sequence and features of the *Trypanosoma brucei* NMT complementation plasmid.
(GBK)

Text S13 **pCMTcNMT.** Sequence and features of the *Trypanosoma cruzi* NMT complementation plasmid.
(GBK)

Text S14 **pCMHsNMT.** Sequence and features of the human NMT2 complementation plasmid.
(GBK)

Text S15 **pCMLmNMT.** Sequence and features of the *Leishmania major* NMT complementation plasmid.
(GBK)

Text S16 **pCMPvPGK.** Sequence and features of the *Plasmodium vivax* PGK complementation plasmid.
(GBK)

Text S17 **pCMSmPGK.** Sequence and features of the *Schistosoma mansoni* PGK complementation plasmid.
(GBK)

Text S18 **pCMTbPGK.** Sequence and features of the *Trypanosoma brucei* PGK complementation plasmid.
(GBK)

Text S19 **pCMTcPGK.** Sequence and features of the *Trypanosoma cruzi* PGK complementation plasmid.
(GBK)

Text S20 **pCMHsPGK.** Sequence and features of the human PGK complementation plasmid.
(GBK)

Text S21 **pCMLmPGKB.** Sequence and features of the *Leishmania major* PGKB complementation plasmid.
(GBK)
