# Supplementary figures and images for: Functional Expression of Parasite Drug Targets and Their Human Orthologs in Yeast
Source: PLoS Negl Trop Dis. 2011 Oct 4;5(10):e1320. doi: 10.1371/journal.pntd.0001320 (PMC3186757; doi:10.1371/journal.pntd.0001320)

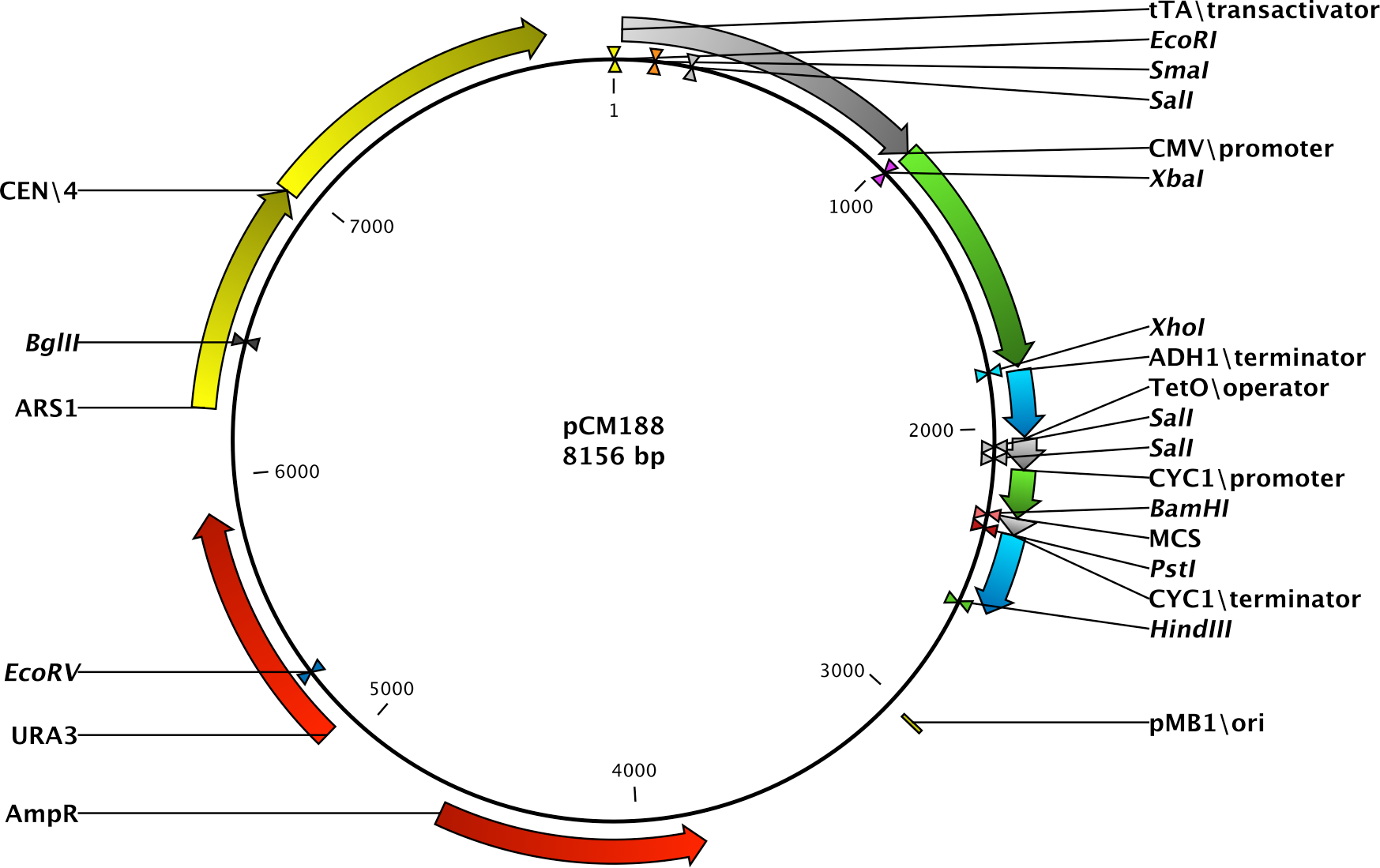

Supplement: Figure S1 — Map of pCM188. Plasmid backbone used for cloning of the cds for heterologous DHFRs, NMTs and PGKs under the control of the tetracycline-regulatable promotor: TetO2. (TIF) [file pntd.0001320.s001.tif]

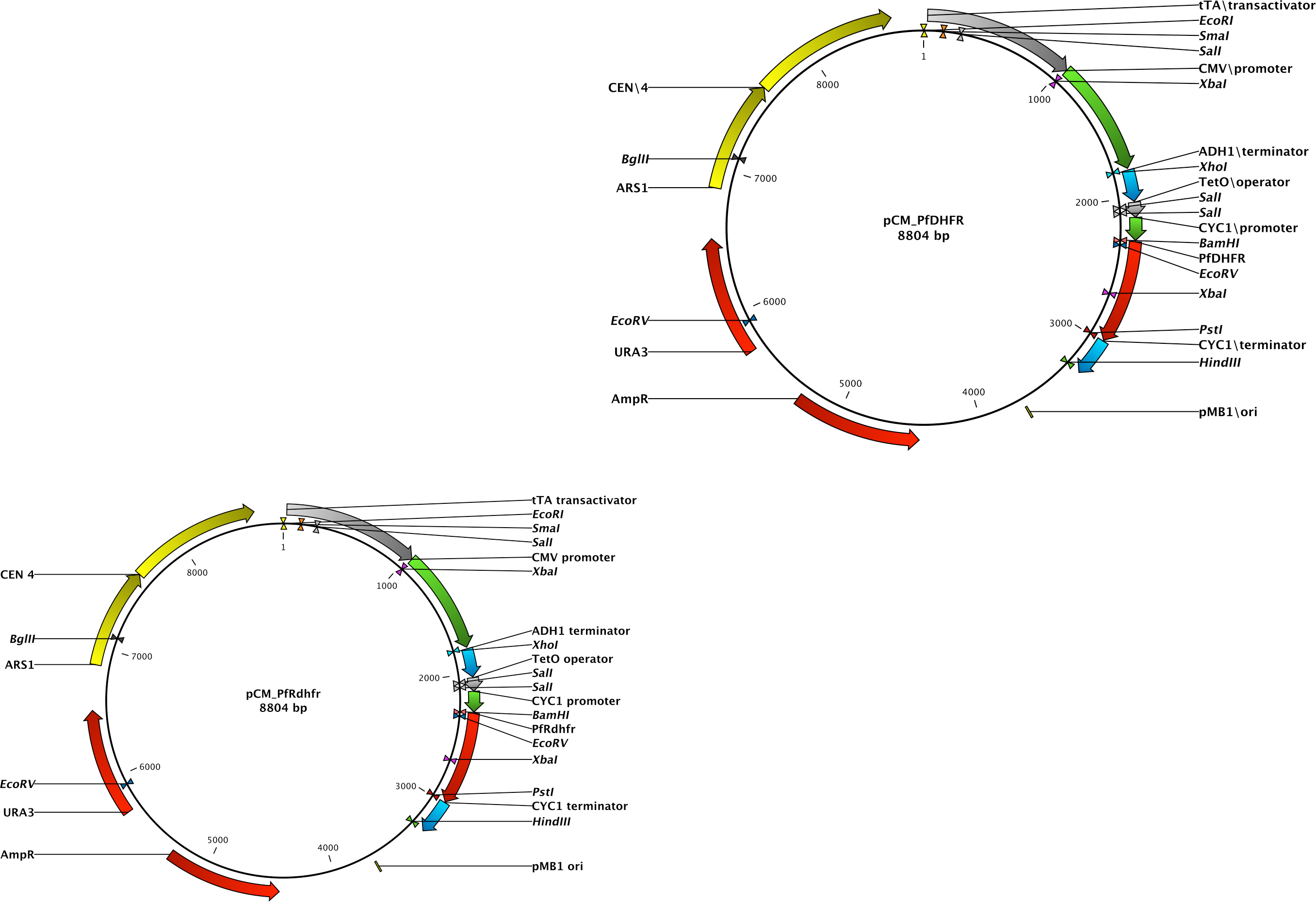

Supplement: Figure S2 — Maps of the Plasmodium falciparum complementation plasmids. Plasmids for expression of cds for heterologous wild-type Plasmodium falciparum DHFR (PfDHFR) and the drug-resistant Plasmodium falciparum DHFR (PfRdhfr) under the control of the TetO2 promoter. (TIF) [file pntd.0001320.s002.tif]

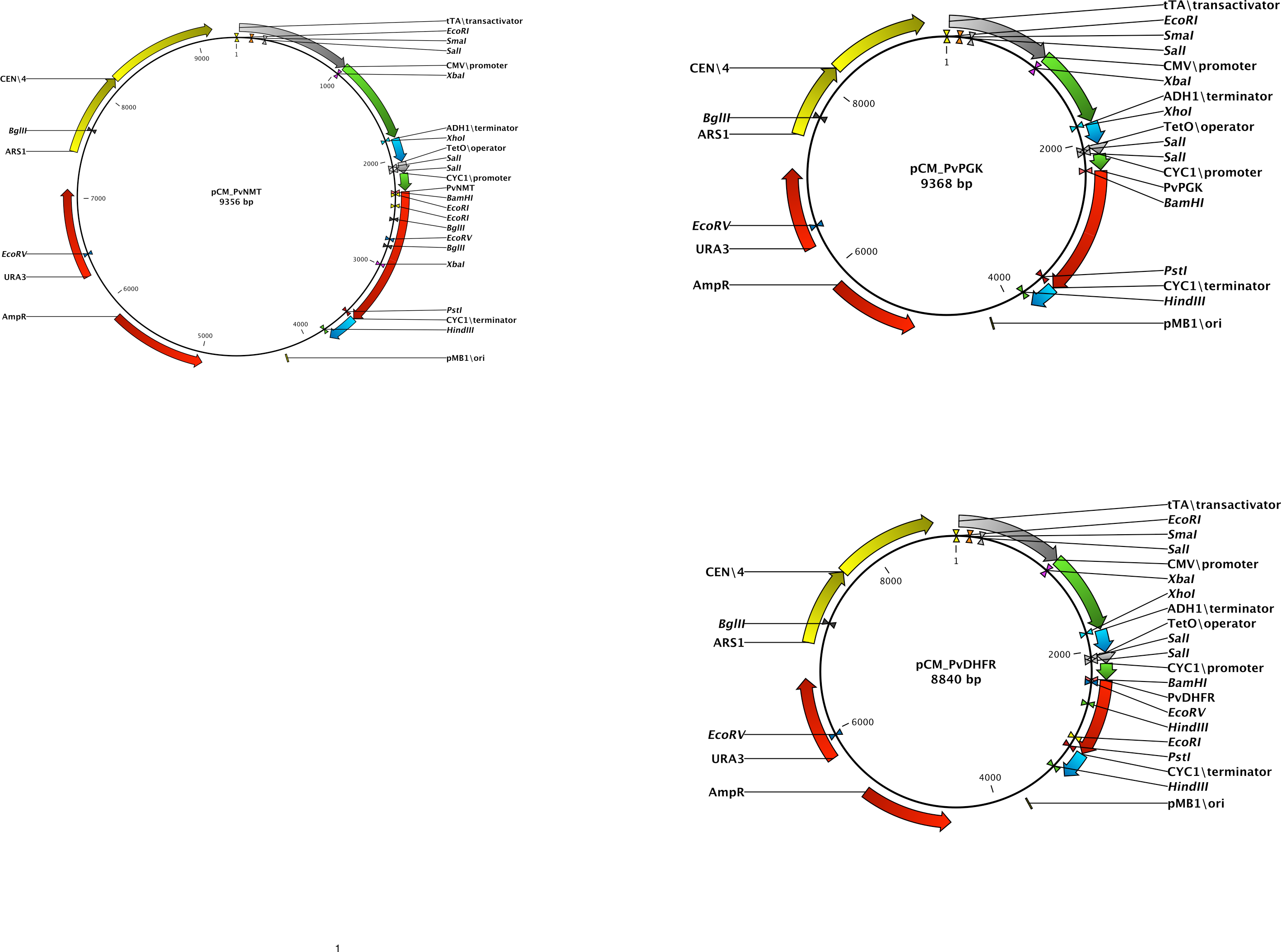

Supplement: Figure S3 — Maps of the Plasmodium vivax complementation plasmids. Plasmids for expression of cds for heterologous Plasmodium vivax DHFR, NMT and PGK under the control of the TetO2 promoter. (TIF) [file pntd.0001320.s003.tif]

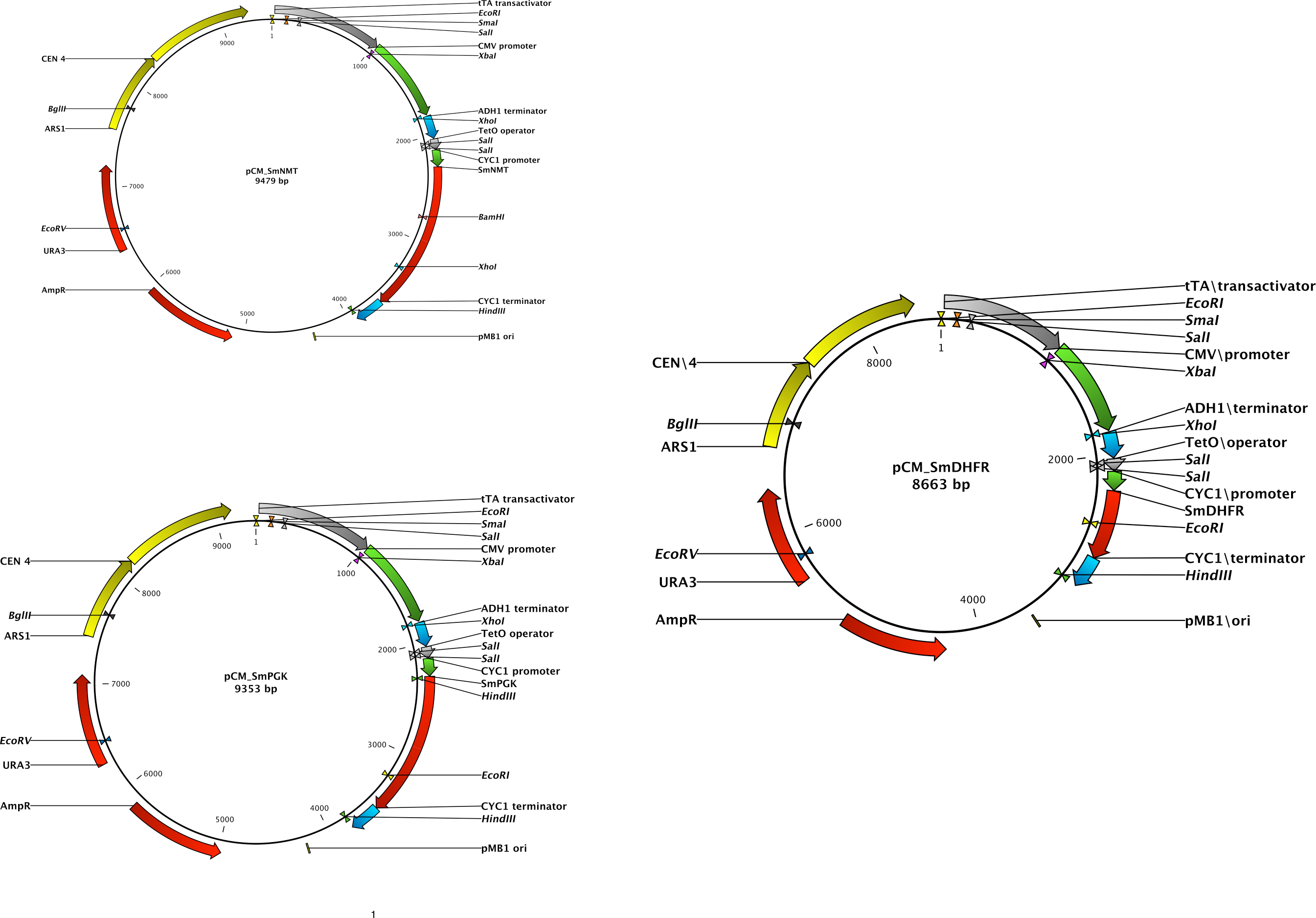

Supplement: Figure S4 — Maps of the Schistosoma mansoni complementation plasmids. Plasmids for expression of cds for heterologous Schistosoma mansoni DHFR, NMT and PGK under the control of the TetO2 promoter. (TIF) [file pntd.0001320.s004.tif]

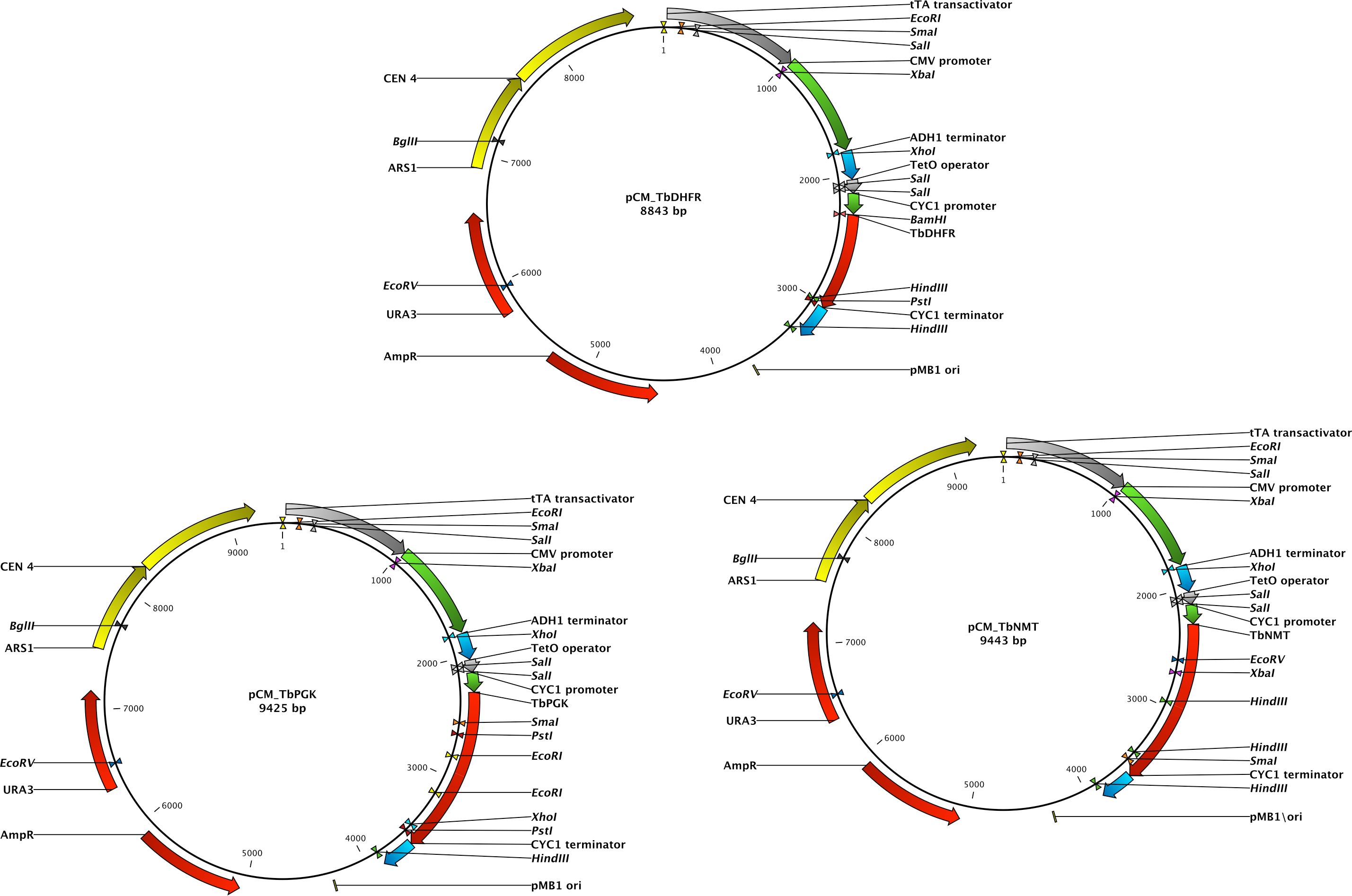

Supplement: Figure S5 — Maps of the Trypanosoma brucei complementation plasmids. Plasmids for expression of cds for heterologous Trypanosoma brucei DHFR, NMT and PGK under the control of the TetO2 promoter. (TIF) [file pntd.0001320.s005.tif]

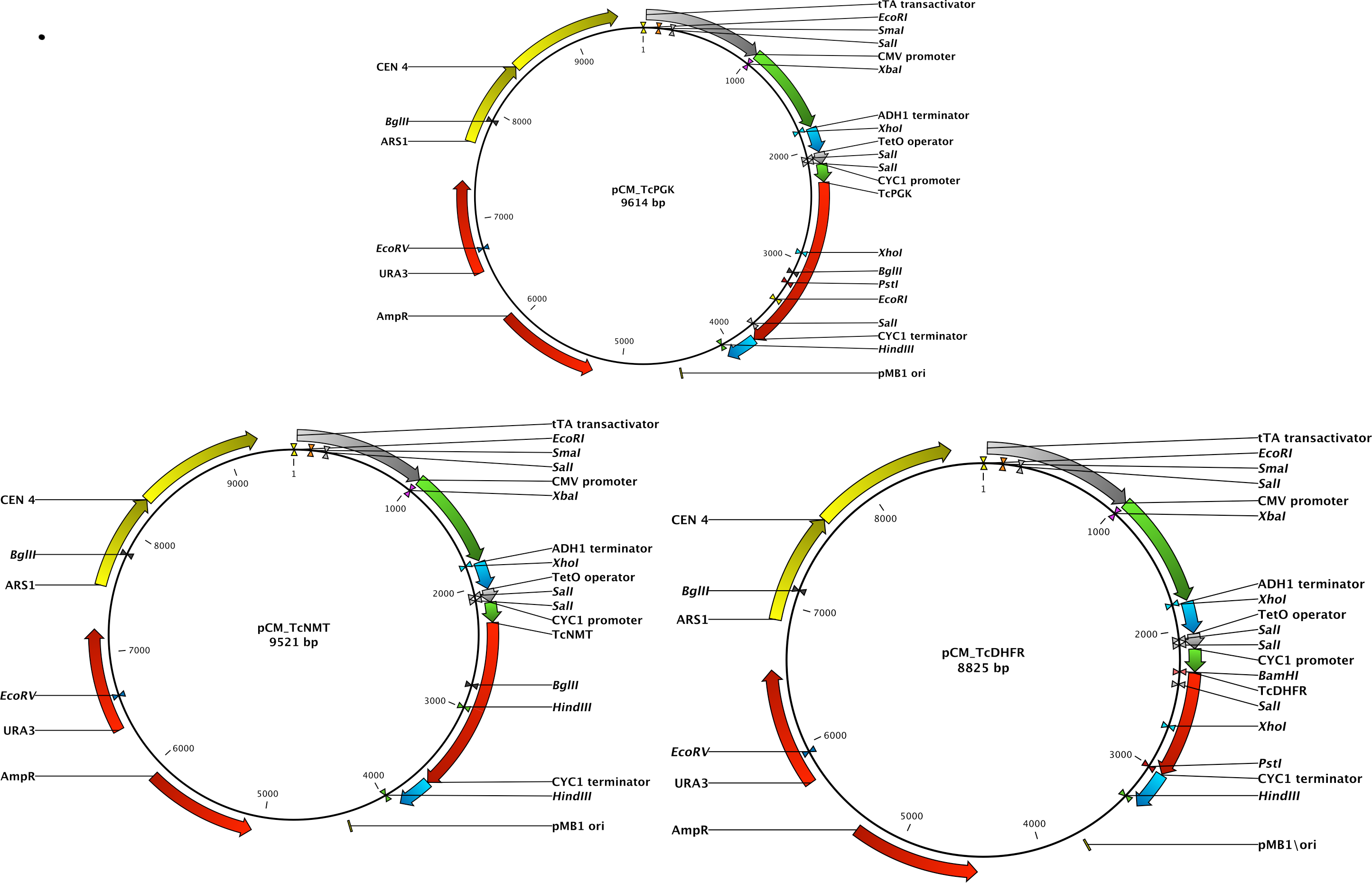

Supplement: Figure S6 — Maps of the Trypanosoma cruzi complementation plasmids. Plasmids for expression of cds for heterologous Trypanosoma cruzi DHFR, NMT and PGK under the control of the TetO2 promoter. (TIF) [file pntd.0001320.s006.tif]

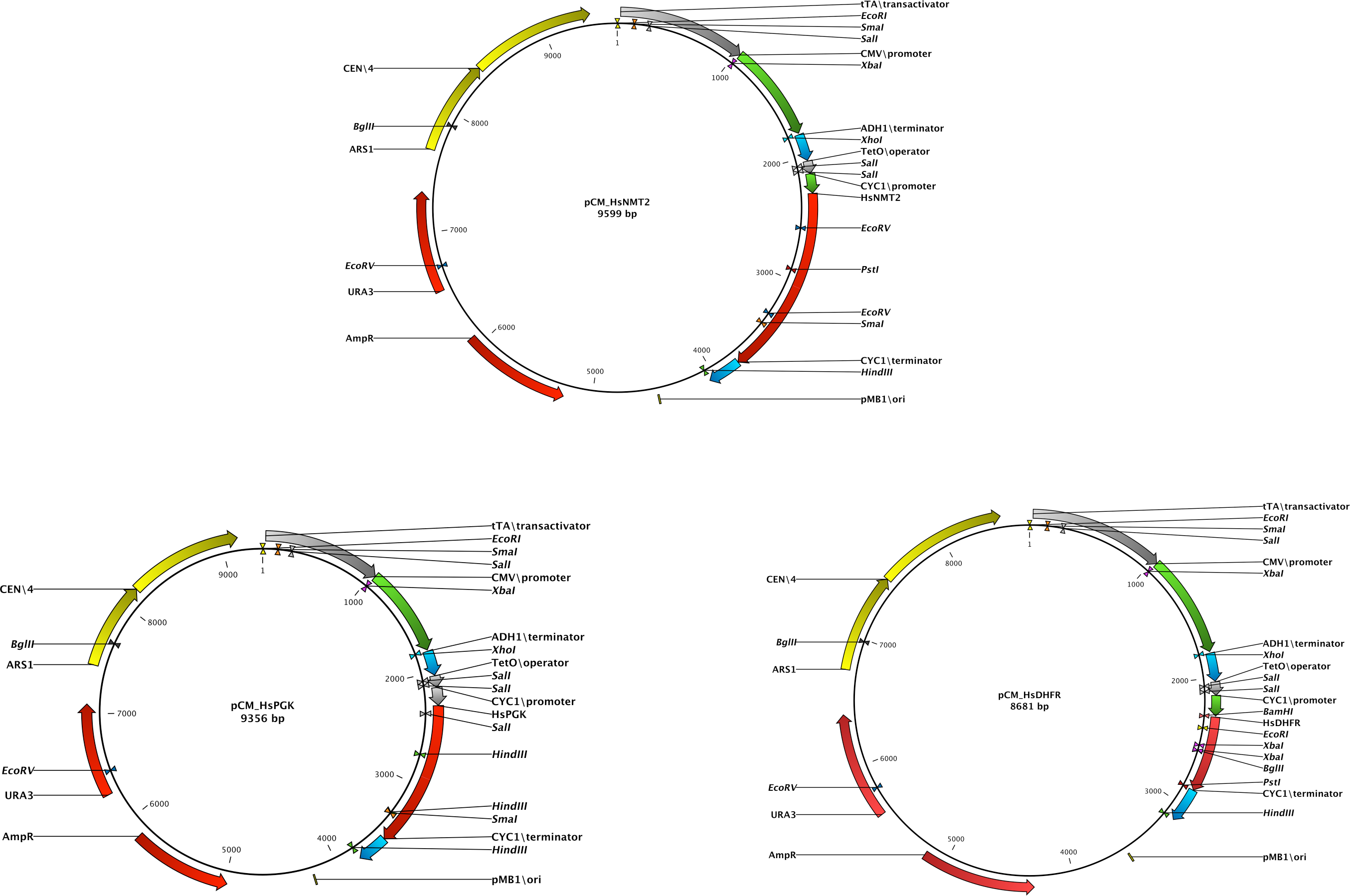

Supplement: Figure S7 — Maps of the human complementation plasmids. Plasmids for expression of cds for heterologous Homo sapiens DHFR, NMT2 and PGK under the control of the TetO2 promoter. (TIF) [file pntd.0001320.s007.tif]

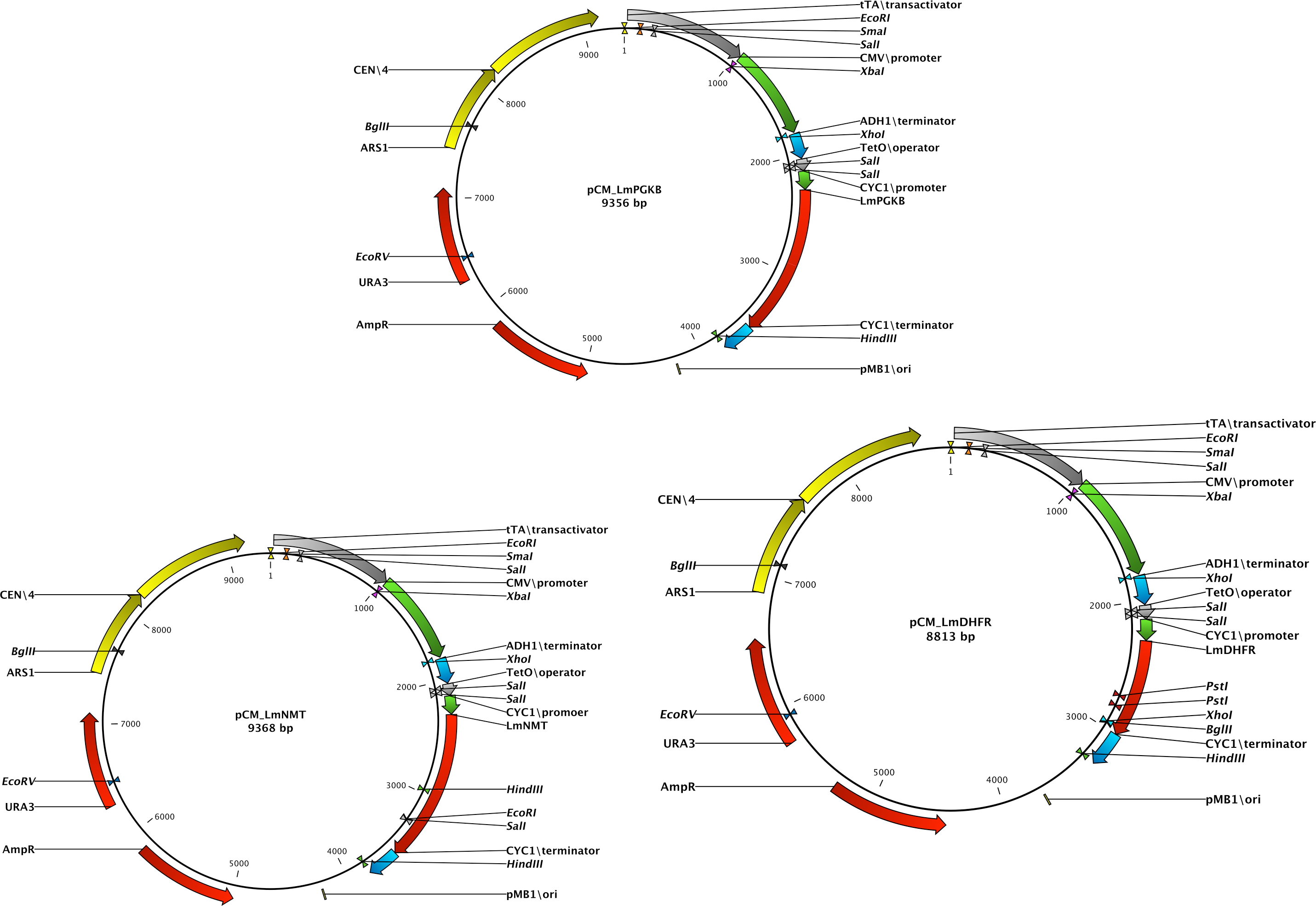

Supplement: Figure S8 — Maps of the Leishmania major complementation plasmids. Plasmids for expression of cds for heterologous Leishmania major DHFR, NMT and PGKB under the control of the TetO2 promoter. (TIF) [file pntd.0001320.s008.tif]

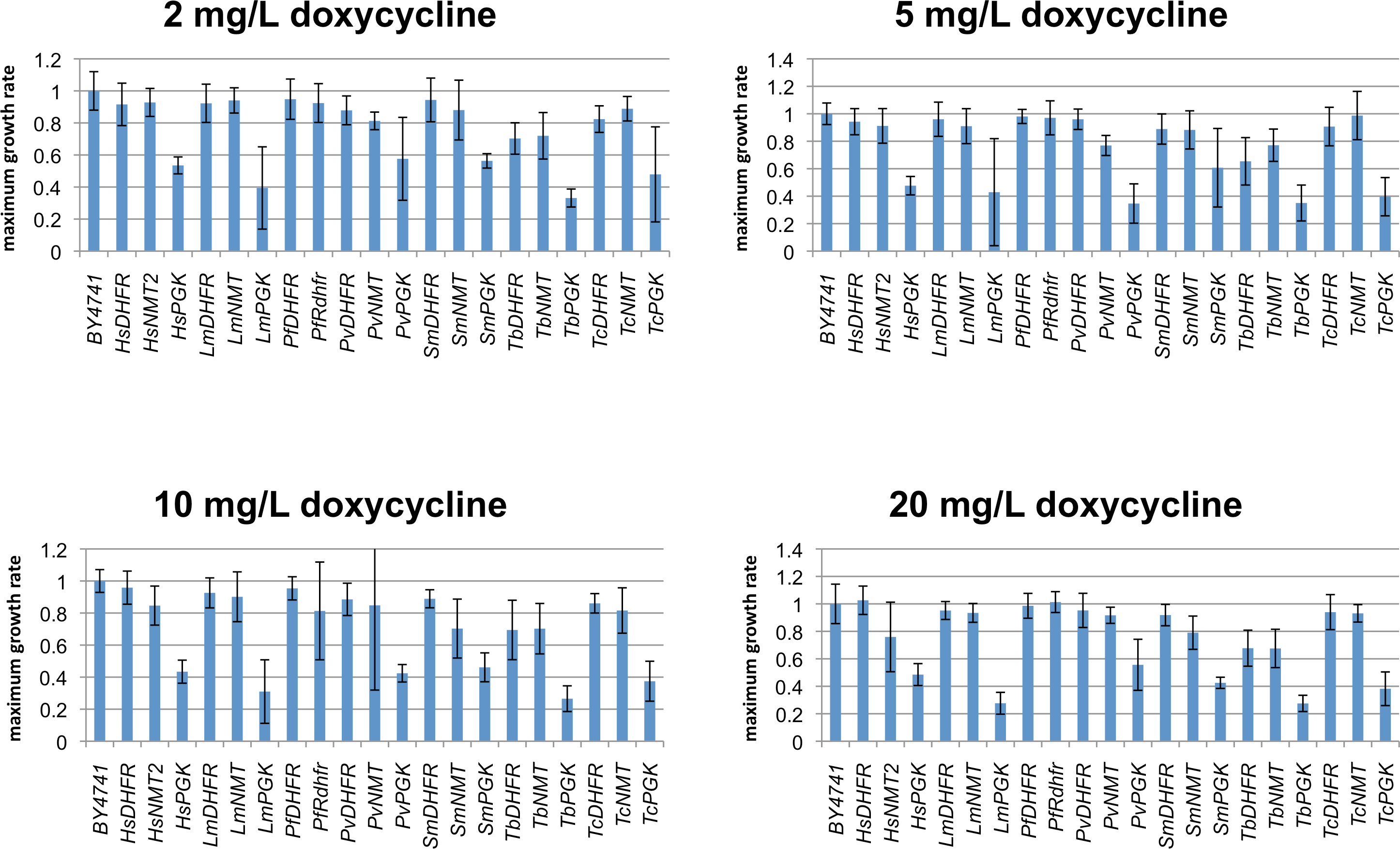

Supplement: Figure S9 — Effect of doxycycline on the maximum growth rate of yeast strains expressing heterologous drug targets. Maximum growth rates (relative to that of the wild type) of yeast strains expressing cds for human or parasite DHFRs, NMTs or PGKs under the control of the TetO2 promoter. (TIF) [file pntd.0001320.s009.tif]

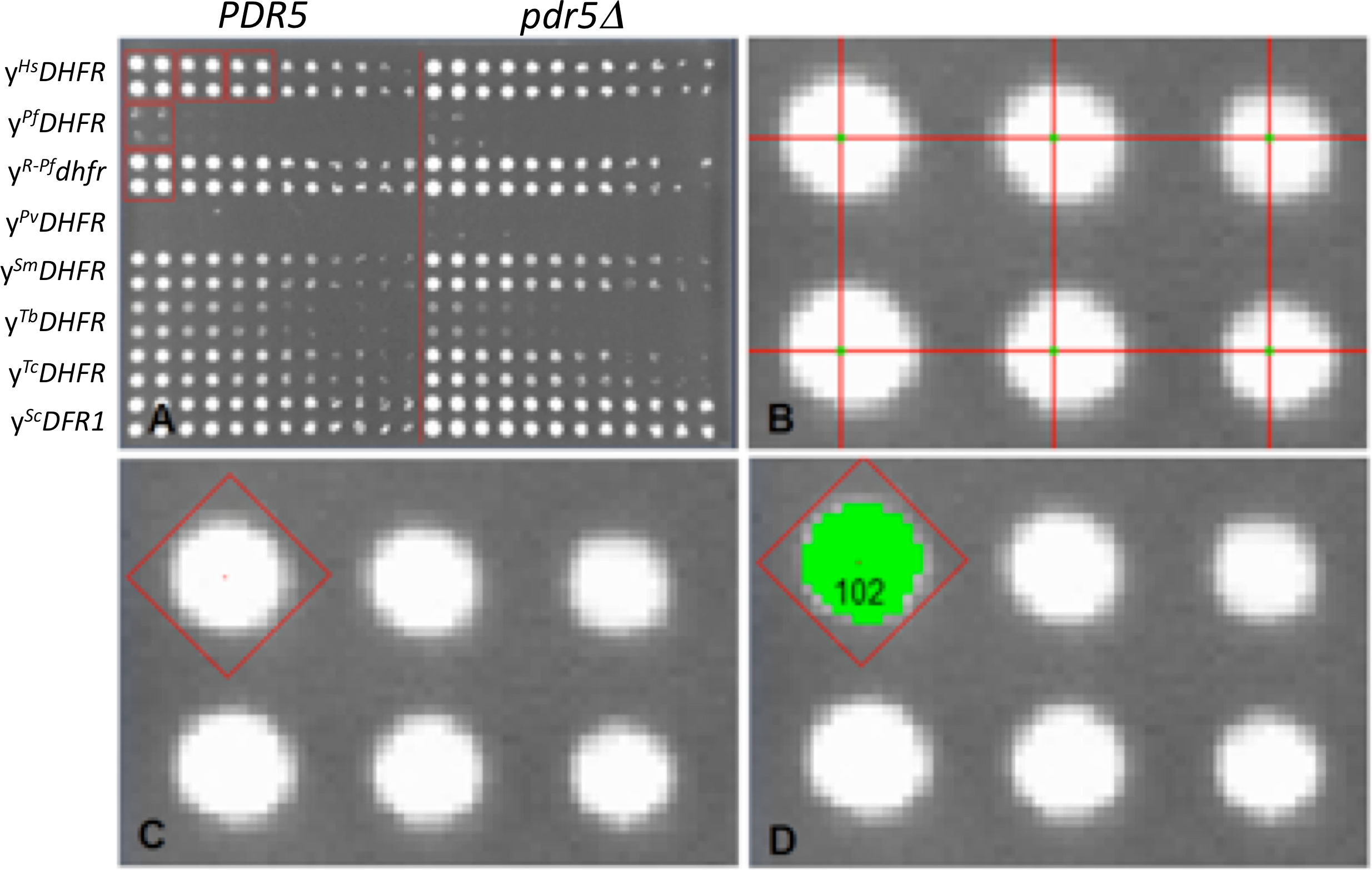

Supplement: Figure S10 — Quantification of the effect of pdr5Δ mutations on pyrimethamine sensitivity. A) Example of plate where serial dilutions of yeast cultures expressing cds for heterologous DHFRs were spotted in quadruplicate onto agar plates containing pyrimethamine and doxycycline. Wild-type PDR5 strains were spotted on the left half of the plate and pdr5Δ deletion mutants on the right half of the plate. B) The intercepts of the brightest row and column of the spots were marked as `colony center. C) The area enclosed by a diamond-shaped frame around the colony center was set as the `colony windo. D) Number of pixels brighter than the threshold within the colony window was set as the `colony sizè, which corresponds to spot's total area. (TIF) [file pntd.0001320.s010.tif]
