# Supplementary material for: Functional Expression of Parasite Drug Targets and Their Human Orthologs in Yeast
Source: PLoS Negl Trop Dis. 2011 Oct 4;5(10):e1320. doi: 10.1371/journal.pntd.0001320 (PMC3186757; doi:10.1371/journal.pntd.0001320)
Supplement: Table S1 — Plasmids used in this study. Details and source of the complementation plasmids used in this work. (DOC) [file pntd.0001320.s011.doc]

| Plasmid name | Features | Derived from | Marker | Source |
| --- | --- | --- | --- | --- |
| pCM188 | CEN plasmid; TetO2 promoter |  | URA3 | EUROSCARF |
| pCM^Pf^DHFR | *Plasmodium falciparum* DHFR | pCM188 | URA3 | This work |
| pCM^PfR^dhfr | Drug resistant *Plasmodium falciparum* dhfr^51I,59R,108N^ | pCM188 | URA3 | This work |
| pCM^Pv^DHFR | *Plasmodium vivax* DHFR | pCM188 | URA3 | This work |
| pCM^Sm^DHFR | *Schistosoma mansoni* DHFR | pCM188 | URA3 | This work |
| pCM^Tb^DHFR | *Trypanosoma brucei* DHFR | pCM188 | URA3 | This work |
| pCM^Tc^DHFR | *Trypanosoma cruzi* DHFR | pCM188 | URA3 | This work |
| pCM^Hs^DHFR | *Homo sapiens* DHFR | pCM188 | URA3 | This work |
| pCM^Sc^DFR1 | *Saccharomyces cerevisiae* DFR1 | pCM188 | URA3 | This work |
| pCM^Lm^DHFR | *Leishmania major* DHFR | pCM188 | URA3 | This work |
| pCM^Pv^NMT | *Plasmodium vivax* NMT | pCM188 | URA3 | This work |
| pCM^Sm^NMT | *Schistosoma mansoni* NMT | pCM188 | URA3 | This work |
| pCM^Tb^NMT | *Trypanosoma brucei* NMT | pCM188 | URA3 | This work |
| pCM^Tc^NMT | *Trypanosoma cruzi* NMT | pCM188 | URA3 | This work |
| pCM^Hs^NMT2 | *Homo sapiens* NMT2 | pCM188 | URA3 | This work |
| pCM^Lm^NMT | *Leishmania major* NMT | pCM188 | URA3 | This work |
| pCM^Pv^PGK | *Plasmodium vivax* PGK | pCM188 | URA3 | This work |
| pCM^Sm^PGK | *Schistosoma mansoni* PGK | pCM188 | URA3 | This work |
| pCM^Tb^PGK | *Trypanosoma brucei* PGK | pCM188 | URA3 | This work |
| pCM^Tc^PGK | *Trypanosoma cruzi* PGK | pCM188 | URA3 | This work |
| pCM^Hs^PGK | *Homo sapiens* PGK | pCM188 | URA3 | This work |
| pCM^Lm^PGKB | *Leishmania major* PGKB | pCM188 | URA3 | This work |
| pCM^Lm^PGKC | *Leishmania major* PGKC | pCM188 | URA3 | This work |
| pCM^Lm^putPGK | *Leishmania major* putative PGK | pCM188 | URA3 | This work |
