# Supplementary material for: Functional Expression of Parasite Drug Targets and Their Human Orthologs in Yeast
Source: PLoS Negl Trop Dis. 2011 Oct 4;5(10):e1320. doi: 10.1371/journal.pntd.0001320 (PMC3186757; doi:10.1371/journal.pntd.0001320)
Supplement: Table S2 — Strains used in this study. Details and source of strains used in this work. (DOC) [file pntd.0001320.s012.doc]

| Strain name | Genotype | Plasmid | Source |
| --- | --- | --- | --- |
| BY4743 | *MAT****a****/MATα his3∆1/his3∆1 leu2∆0/leu2∆0 met15∆0/MET15 LYS2/lys2∆0 ura3∆0/ura3∆0* |  | EUROSCARF |
| BY4741 | *MAT****a*** *his3∆1 leu2∆0 met15∆0 LYS2 ura3∆0* |  | EUROSCARF |
| *dfr1∆*/+ | *dfr1∆::KanMX/DFR1* in BY4743 |  | EUROSCARF |
| *dfr1∆*/+ *pdr5∆*/+ | *dfr1∆::KanMX/DFR1 pdr5∆::HisMX/PDR5* in BY4743 |  | This work |
| *y^Pf^DHFR* | *dfr1∆::KanMx his3∆1 leu2∆0 MET15 lys2∆0 MATα* | pCM*^Pf^DHFR* | This work |
| *y^PfR^dhfr* | *dfr1∆::KanMx his3∆1 leu2∆0 MET15 lys2∆0 MAT****a*** | pCM*^PfR^dhfr* | This work |
| *y^Pv^DHFR* | *dfr1∆::KanMx his3∆1 leu2∆0 MET15 lys2∆0 MATα* | pCM*^Pv^DHFR* | This work |
| *y^Sm^DHFR* | *dfr1∆::KanMx his3∆1 leu2∆0 MET15 lys2∆0 MATα* | pCM*^Sm^DHFR* | This work |
| *y^Tb^DHFR* | *dfr1∆::KanMx his3∆1 leu2∆0 MET15 lys2∆0 MATα* | pCM*^Tb^DHFR* | This work |
| *y^Tc^DHFR* | *dfr1∆::KanMx his3∆1 leu2∆0 MET15 lys2∆0 MATα* | pCM*^Tc^DHFR* | This work |
| *y^Hs^DHFR* | *dfr1∆::KanMx his3∆1 leu2∆0 MET15 lys2∆0 MATα* | pCM*^Hs^DHFR* | This work |
| *y^Sc^DFR1* | *dfr1∆::KanMx his3∆1 leu2∆0 MET15 lys2∆0 MATα* | pCM*^Sc^DFR1* | This work |
| *y^Pf^DHFR_p* | *dfr1∆::KanMx pdr5∆::HisMX his3∆1 leu2∆0 MET15 lys2∆0 MATα* | pCM*^Pf^DHFR* | This work |
| *y^PfR^dhfr_p* | *dfr1∆::KanMx pdr5∆::HisMX his3∆1 leu2∆0 MET15 lys2∆0 MATα* | pCM*^PfR^dhfr* | This work |
| *y^Pv^DHFR_p* | *dfr1∆::KanMx pdr5∆::HisMX his3∆1 leu2∆0 MET15 lys2∆0 MATα* | pCM*^Pv^DHFR* | This work |
| *y^Sm^DHFR_p* | *dfr1∆::KanMx pdr5∆::HisMX his3∆1 leu2∆0 MET15 lys2∆0 MATα* | pCM*^Sm^DHFR* | This work |
| *y^Tc^DHFR_p* | *dfr1∆::KanMx pdr5∆::HisMX his3∆1 leu2∆0 MET15 lys2∆0 MATα* | pCM*^Tc^DHFR* | This work |
| *y^Tb^DHFR_p* | *dfr1∆::KanMx pdr5∆::HisMX his3∆1 leu2∆0 MET15 lys2∆0 MATα* | pCM*^Tb^DHFR* | This work |
| *y^Hs^DHFR_p* | *dfr1∆::KanMx pdr5∆::HisMX his3∆1 leu2∆0 MET15 lys2∆0 MATα* | pCM*^Hs^DHFR* | This work |
| *y^Sc^DFR1_p* | *dfr1∆::KanMx pdr5∆::HisMX his3∆1 leu2∆0 MET15 lys2∆0 MATα* | pCM*^Sc^DFR1* | This work |
| *y^Pv^PGK_p* | *pgk1∆::KanMx pdr5∆::HisMX his3∆1 leu2∆0 MET15 lys2∆0 MATα* | pCM*^Pv^PGK* | This work |
| *y^Sm^PGK_p* | *pgk1∆::KanMx pdr5∆::HisMX his3∆1 leu2∆0 MET15 lys2∆0 MATα* | pCM*^Sm^PGK* | This work |
| *y^Tc^PGK_p* | *pgk1∆::KanMx pdr5∆::HisMX his3∆1 leu2∆0 MET15 lys2∆0 MATα* | pCM*^Tc^PGK* | This work |
| *y^Tb^PGK_p* | *pgk1∆::KanMx pdr5∆::HisMX his3∆1 leu2∆0 MET15 lys2∆0 MATα* | pCM*^Tb^PGK* | This work |
| *y^Hs^PGK_p* | *pgk1∆::KanMx pdr5∆::HisMX his3∆1 leu2∆0 MET15 lys2∆0 MATα* | pCM*^Hs^PGK* | This work |
| *Y^Lm^PGK_p* | *pgk1∆::KanMx pdr5∆::HisMX his3∆1 leu2∆0 MET15 lys2∆0 MATα* | pCM*^Lm^PGK* | This work |
| *y^Pv^NMT_p* | *nmt1∆::KanMx pdr5∆::HisMX his3∆1 leu2∆0 met15∆0 LYS2 ura3∆0 MAT****a*** | pCM*^Pv^NMT* | This work |
| *y^Sm^NMT_p* | *nmt1∆::KanMx pdr5∆::HisMX his3∆1 leu2∆0 met15∆0 LYS2 ura3∆0 MAT****a*** | pCM*^Sm^NMT* | This work |
| *y^Tc^NMT_p* | *nmt1∆::KanMx pdr5∆::HisMX his3∆1 leu2∆0 met15∆0 LYS2 ura3∆0 MAT****a*** | pCM*^Tc^NMT* | This work |
| *y^Tb^NMT_p* | *nmt1∆::KanMx pdr5∆::HisMX his3∆1 leu2∆0 met15∆0 LYS2 ura3∆0 MAT****a*** | pCM*^Tb^NMT* | This work |
| *y^Hs^NMT_p* | *nmt1∆::KanMx pdr5∆::HisMX his3∆1 leu2∆0 met15∆0 LYS2 ura3∆0 MAT****a*** | pCM*^Hs^NMT* | This work |
| *y^Lm^NMT_p* | *nmt1∆::KanMx pdr5∆::HisMX his3∆1 leu2∆0 met15∆0 LYS2 ura3∆0 MAT****a*** | pCM*^Lm^NMT* | This work |
